# A Window on Maize Evolution

**DOI:** 10.1371/journal.pbio.1000411

**Published:** 2010-06-29

**Authors:** Mary Hoff

**Affiliations:** Freelance Science Writer, Stillwater, Minnesota, United States of America

**Figure pbio-1000411-g001:**
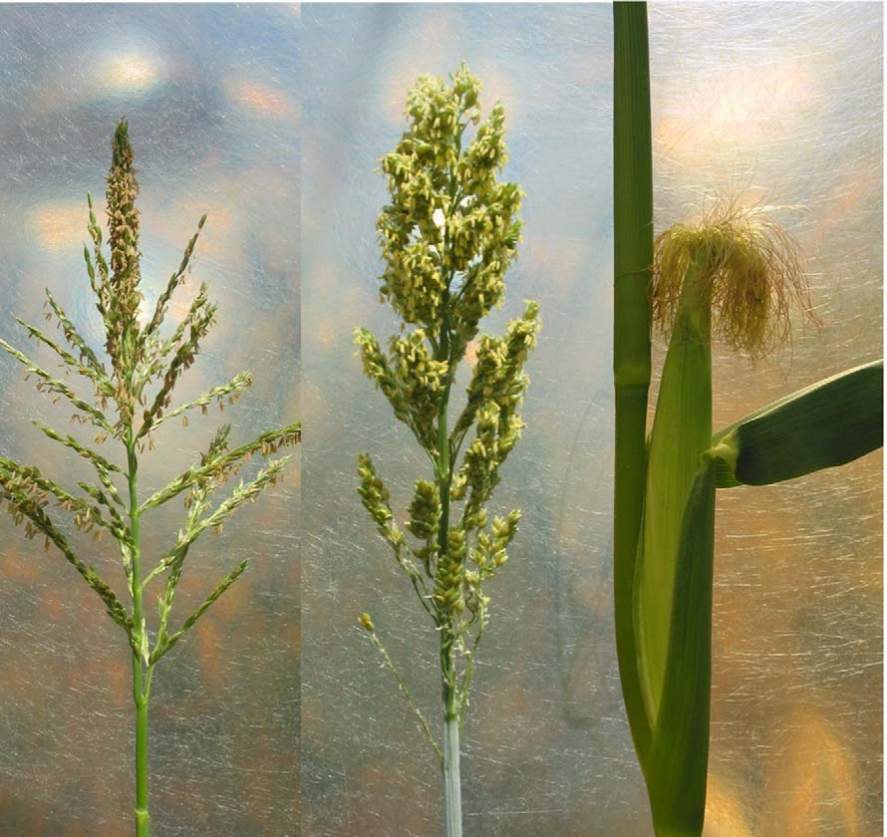
The genome of maize is the result of a whole-genome duplication that created two duplicate genomes—each orthologous to the entire sorghum genome—that have been reduced by fractionation. This process of fractionation can be seen as analogous to the evolution of inflorescence between the two species, with the perfect flowers of sorghum's single inflorescence fractionating into imperfect male and female flowers on separate inflorescences in maize. Left panel: maize male flower; middle panel: sorghum flower; right panel: ear of maize topped with female tassels.

[Fig pbio-1000411-g001]Evolution is iffy business. Between one generation and the next, the millions or billions of base pairs that make up an organism's genetic material go through subtle but sometimes significant changes, with bits added, deleted, and moved around in the off chance that the new combination may serve its owner better than the previous one did its parents. Clearly some do—we are here, and so are nematodes, giraffes, jellyfish, and dandelions, all cut from the same cloth and shaped by such random alterations. But far many more do not. The road to biodiversity is littered with the fleeting memory of genetic remixes that rendered their short-lived owners incapable of survival.

But what if an organism had two sets of chromosome pairs—one to provision it with the basic functions it needs to stay alive, and the other to play around with, evolutionarily? If a new combination in the doubled assemblage provided an adaptive advantage, so much the better. If not, the process of trying it out would likely not be lethal, thanks to the backup provided by the second chromosome set.

It turns out that's just the game that many lineages of eukaryotes, especially plants, have up their genome sleeves. Somewhere in the course of their evolutionary history, maize, wheat, and a number of other species underwent a process that left them polyploid, or in possession of multiple pairs of chromosomes, rather than just the single pair most animals own. These extra pairs tend to make them bigger and, from the human perspective, more productive. They also provide their owners with more raw material for evolution, allowing them to undergo major alterations in genetic composition without the risk of losing fundamental function.

Just how plants let evolution play with this extra set of genes has been the subject of study in *Arabidopsis*, a little dicot with the big distinction of being the white rat, research-wise, of the plant world. *Arabidopsis* is also multiply tetraploid, but has evolved to act like a diploid. Four years ago, researchers made a mechanism-mystifying discovery: this species' two sets of chromosomes were differentially altered, or fractionated, with more of the modifications accumulating on one set than the other. This selectivity is both adaptive—it keeps the original pair intact, providing a safety net during experimentation as the other homeolog (matched chromosome) gradually sheds and recombines genetic material, ridding excess and creating new combinations—and mystifying. How can change be directed toward one set, but not the other? Now, the same laboratory that mystified us with *Arabidopsis* genome research is back again.

University of California Berkeley, plant biologists Michael Freeling, Margaret Woodhouse, James Schnable, and colleagues set out to search for the answer by comparing two recently sequenced grasses—sorghum and B73 inbred maize. Shortly after the two species split off from a common ancestor 12 million years ago, maize became tetraploid. Clues to what it has and hasn't done with the extra set of genes created in the process lie hidden in the similarities and differences between the two species today.

The researchers homed in on 37 stretches of sorghum DNA containing 2,943 shared genes to use as a “before evolution” proxy picture with which to compare the “after evolution” current maize genome. They found that 43% of the genes were retained to at least some major extent in maize, with a disproportionate share of the retained genes encoding transcription factors. When they looked for stretches of genetic material still present in sorghum but absent or altered in maize, they found that—like *Arabidopsis*—corn exhibited biased fractionation, with change occurring 2.3 times more frequently on one of the homeologs as on the other. Most of the changes were gene deletions rather than relocations, and most occurred on a gene-by-gene basis rather than in clusters. The homeolog with the bulk of the changes was also more likely to be missing transposons and other dispensible DNA making up the bulk of the genome. These researchers discovered that this DNA in between genes was even more likely to be differentially fractionated than were genes themselves.

Comparing the gene remnants in maize with both sorghum and rice (another grass), the researchers discovered that sequences that had been deleted often have short, identical sequences on both sides of them in the inferred progenitor. This suggests that the mechanism for fractionation here is primarily “illegitimate recombination”—a type of sequence removal in which nearby identical sequences line up, creating a loop out of the base pairs between them that eventually pinches off.

What makes one homeolog preferentially fractionate? As with so many other mysterious happenings with genetic material, epigenetic changes came quickly to mind as a likely suspect, so the researchers checked whether the phenomenon was associated with the presence of methylated domains. Though it was not, it's still possible that another type of epigenetic mark, such as histone modification, might well be at play.

The authors note that, from an evolutionary standpoint, biased fractionation makes lots of sense. A mutation that inactivates one homeolog automatically puts strong selective pressure against a debilitating mutation on the other. On the other hand, continued mutations on the already inactivated chromosome can't do any more harm. Eventually, as the forces of selection (natural or otherwise) act, the tetraploid rids itself of extra DNA and generates new combinations and juxtapositions with which to face its ever-changing environment and, in maize's case, meet ever-growing demands to provide food and fuel to a fast-growing human population.

**Woodhouse MR, Schnable JC, Pedersen BS, Lyons E, Lisch D, et al. (2010) Following Tetraploidy in Maize, a Short Deletion Mechanism Removed Genes Preferentially from One of the Two Homeologs. doi:10.1371/journal.pbio.1000409**

